# Palladium nanoclusters as a label to determine GFAP in human serum from donors with stroke by bimodal detection: inductively coupled plasma-mass spectrometry and linear sweep voltammetry

**DOI:** 10.1007/s00604-023-06059-5

**Published:** 2023-11-30

**Authors:** Alejandro Rodríguez-Penedo, Estefanía Costa-Rama, Beatriz Fernández, Carmen García-Cabo, Lorena Benavente, Sergio Calleja, M. Teresa Fernández-Abedul, Rosario Pereiro

**Affiliations:** 1 Department of Physical and Analytical Chemistry, University of Oviedo, Julian Clavería 8, 33006 Oviedo, Spain; 2 Department of Neurology, Hospital Universitario Central de Asturias (HUCA), Oviedo, Spain

**Keywords:** Palladium nanoclusters, Glial fibrillary acidic protein, Competitive immunoassay, ICP-MS, Linear sweep voltammetry

## Abstract

Water-soluble, stable, and monodisperse palladium nanoclusters (PdNCs) were synthesized using NaBH_4_ as a reductant and lipoic acid as a ligand. PdNCs, measured by high-resolution transmission electron microscopy, showed a round shape and a diameter of 2.49 ± 0.02 nm. It was found that each PdNC contains 550 Pd atoms on average. These PdNCs offer high amplification as a label of biochemical reactions when inductively coupled plasma-mass spectrometry (ICP-MS) is used as a detector. In addition, PdNCs have catalytic activity on electrochemical reactions, allowing detection by linear sweep voltammetry (LSV). As a proof of applicability, a competitive immunoassay based on PdNC labels was developed for the determination of glial fibrillary acidic protein (GFAP) in human serum, comparing ICP-MS and LSV detection. GFAP is a biomarker for differentiating between patients with ischemic stroke (IS) and hemorrhagic stroke (HS). The limit of detection (LoD), corresponding to IC_10_ (4-parameter logistic curve), was 0.03 pM of GFAP, both by ICP-MS and LSV, being lower than the 0.31 pM LoD provided by the ELISA commercial kit. Using the error profile method, 0.03 pM and 0.11 pM LoDs were obtained respectively by ICP-MS and LSV: LoD is lower by ICP-MS due to the better precision of the measurements. The analyses of human serum samples from IS, HS, and control (CT) donors using PdNC labels and detection by ICP-MS and LSV were validated with a commercial ELISA kit (for CT donors only ICP-MS provided enough sensitivity). Results point out toward the future use of PdNCs as a label in other immunoprobes for the determination of specific proteins requiring very low LoDs as well as the development of electrochemical decentralized methodologies.

**Graphical Abstract:**

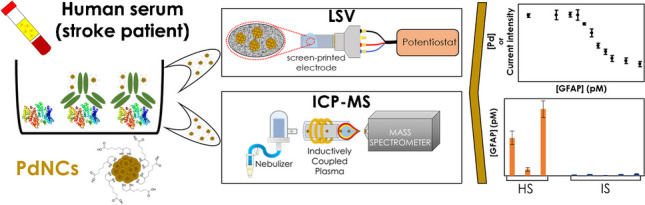

**Supplementary Information:**

The online version contains supplementary material available at 10.1007/s00604-023-06059-5.

## Introduction

Stroke is a major public health concern and a leading cause of long-term disability and mortality worldwide. It occurs when the blood supply to the brain is disrupted, resulting in the death of brain cells and the loss of neurological function. Clinically, strokes are categorized into two types: ischemic stroke (IS) and hemorrhagic stroke (HS). Ischemic stroke accounts for approximately 87% of all strokes, while hemorrhagic stroke represents the remaining percentage. However, these proportions can vary across countries [1]. Currently, clinical differentiation between the two types of strokes is typically carried out using computerized axial tomography, which is unavailable in pre-hospital settings. A different treatment is applied depending on the type of the stroke, making necessary its fast identification. Mortality rates and the occurrence of permanent sequelae are greatly reduced if suitable treatment is started promptly [2, 3]. Consequently, there is great interest in the development of analytical strategies to differentiate between IS and HS [4, 5].

Glial fibrillary acidic protein (GFAP), a brain-specific protein, is released into the bloodstream after brain tissue damage. Within 2–6 h after symptom onset, a significant difference in serum GFAP levels has been observed between hemorrhagic and ischemic acute strokes [6, 7]. Therefore, GFAP is considered a biomarker for differentiating between patients within the acute phase of stroke symptom onset [8–10]. Additionally, GFAP levels might be associated with stroke severity, infarct volumes, bleeding volumes, and stroke location [11, 12]. Serum and blood samples have been evaluated for GFAP determination; however, serum levels of GFAP were reported to be undetectable in some donors with IS as well as in individuals without stroke (Control, CT) [7, 13]. It is worth noting that GFAP concentrations reported in the literature exhibit considerable variation, influenced by factors such as the time elapsed after the stroke episode and the race of the patients [6, 10, 12, 13].

Enzyme-linked immunosorbent assays (ELISA) are widely used in biomedical research and clinical diagnostics to determine the concentration of specific analytes [14]. Even so, they have certain limitations related to the color changes over time (which may lead to false positives) and limited sensitivity. In the case of GFAP, the sensitive single molecule array (SIMOA) technology has been recently proposed for the detection of GFAP with higher sensitivity than conventional ELISA [15]. Nonetheless, the development of highly sensitive analytical techniques remains an ongoing area of interest, particularly for the accurate determination of GFAP and other biomarkers present at low concentrations in clinical samples.

In recent years, nanotechnology has emerged as a powerful tool to develop sensitive methods for biomedical and biosensing applications. Among different nanomaterials, metal nanoclusters (MNCs) have attracted considerable attention [16]. The small size of MNCs (with a diameter below 3 nm) makes them particularly suitable for labelling of biomolecules in immunoassays since they will have a lower risk of hindering the antibody (Ab) recognition capabilities. MNCs have shown a high potential as labels for the detection of biomolecules by inductively coupled plasma-mass spectrometry (ICP-MS) [17]. They are composed of a unique given metal type and contain many atoms of such metal, thus allowing high signal amplification: detection of target biomolecules even at the low attogram level has been reported [18]. MNCs are usually synthesized with Au, Ag, or Pt [19–21], although alternative metals such as Ir or Pd have been recently proposed [22, 23]. The use of PdNCs is particularly interesting because they offer the possibility to be detected not only by ICP-MS, but also with electrochemical techniques. PdNCs have catalytic activity on multiple electrochemical reactions of analytical interest, such as the oxygen reduction reaction (ORR) and the hydrogen evolution reaction (HER) [23, 24]. The simplicity and low cost of the equipment make these electrochemical techniques very adequate for on-site analysis. Although it has not been explored up to date, the applicability of a unique metal label that allows bimodal detection of target analytes by elemental MS and electrochemical techniques could be of interest in clinical settings.

In this study, we proposed for the first time PdNCs as a highly sensitive label for bimodal determination by ICP-MS and linear sweep voltammetry (LSV). ICP-MS is considered a highly sensitive analytical technique providing multi-elemental capabilities, while LSV allows an easy adaptation to the point-of-care (POC) analysis due to both the low cost of the equipment and the simplicity of adapting it to decentralized analysis. As a proof of concept, a competitive immunoassay based on the use of PdNCs as a label for the determination of GFAP in human serum is here proposed to differentiate between CT, IS, and HS groups. With this aim, the synthesis of monodisperse and electrocatalytic PdNCs was first pursued. Then, sample preparation and immunoassay steps were optimized to perform ICP-MS and LSV measurements with the same sample, comparing the analytical characteristics of the methodologies. Both detection systems were applied to real sample analysis. To validate the GFAP content obtained using PdNC labels, we also employed a commercial ELISA kit for the same samples, in cases where the sensitivity of the commercial kit was sufficiently high.

## Experimental

### Materials and reagents

The synthesis of PdNCs was carried out using potassium tetrachloropalladate (purity ≥ 99.99%, Sigma-Aldrich, USA) as a precursor salt. The selected ligand agent was DL-α-lipoic acid (purity ≥ 98%, ACROS Organics, China), and sodium borohydride (purity > 98%, Sigma-Aldrich, USA) was used as the reducing agent. The basic solution was prepared using sodium hydroxide (purity > 98%, Sigma-Aldrich, USA). Amicon Ultra-0.5 filters (membrane PLBC, 3 kDa and 100 kDa, Millipore Amicon, Ireland) were employed for purification. Labelling of the antibodies with PdNCs was performed with 1-ethyl-3-(3-dimethylaminopropyl) carbodiimide (EDC) (98% powder; Acros Organics) and N-hydroxysuccinimide (NHS) (> 98% powder; Sigma-Aldrich).

Concerning the immunoassays, the anti-human-GFAP antibody (anti-hGFAP, produced in rabbit, Sigma-Aldrich, ab68428) and a recombinant protein fragment of Human GFAP (derived from Streptococcal protein G, Sigma-Aldrich, APREST85954) were used both to evaluate the recognition capabilities of the ab labelled with PdNCs and to perform the competitive immunoassay for GFAP determination in human serum samples. Microtiter plates (96 well; Thermo Fisher Scientific, Germany) were used. The plate was coated with the recombinant protein fragment of Human GFAP and blocked with bovine serum albumin (BSA) (99% powder, Merck, Germany). Phosphate buffer (PB) solution (pH = 7.4) was made using anhydrous disodium hydrogen phosphate and sodium dihydrogen phosphate dihydrate (both 100% pure, VWR, Belgium). PB solution (10 mM) with 0.05% of Tween-20 (Sigma-Aldrich) was employed in the immunoassay procedure. Furthermore, sodium dodecyl sulfate (SDS) (≥ 99%, Sigma-Aldrich) and β-mercaptoethanol (> 99%, Sigma-Aldrich) were evaluated to extract PdNCs from the wells. To validate the methodologies based on PdNC labels, the GFAP ELISA kit (Abcam, ab222279) was used.

For ICP-MS measurements, Pd and Rh standards were prepared from 1000 mg L^−1^ Pd and Rh standard solutions (Sigma-Aldrich) in 2% ultrapure HNO_3_ (65%, Merck, Germany). For electrochemical measurements, HNO_3_ solutions (0.1–1 M) were employed for optimization studies. Deionized ultrapure water, resistivity 18.2 MΩ·cm (Purelab Flex 3&4; ELGA-Veolia, High Wycombe), was utilized throughout the experiments.

### Instrumentation

A high-resolution transmission electron microscope (HR-TEM) (LIBRA 200 FE, Carl Zeiss) with an energy dispersive X-ray (EDX) spectrometer for microanalysis allowed morphological characterization of the PdNCs. A spectrophotometer (Cary 60 UV–VIS, Agilent Technologies, USA) was employed for absorption measurements of the synthesis precursors and PdNC suspensions. A Suprasil quartz cuvette (model 114F-QS, Sigma-Aldrich) was employed for such purpose, with an optical path of 10 mm and a chamber volume of 3 mL.

ICP-MS measurements were performed using a 7900 series quadrupole ICP-MS (Agilent Technologies, USA). ICP-MS analyses were carried out to calculate the Pd concentration in PdNC synthesis and to determine the GFAP after the competitive immunoassay by measuring the Pd in each well. Operation parameters employed for ICP-MS analysis are collected in Table S1 (*Electronic Supplementary Material, ESM)*.

Electrochemical measurements were performed using a µ-Autolab Type II (EcoChemie BV, Utrecht, Netherlands) controlled by the Autolab GPES software. Flexible screen-printed electrodes (SPCEs, MicruX Technologies, Spain) with working (7.1 mm^2^) and counter electrodes made of C ink and a pseudoreference made of Ag ink were employed. Additionally, a box connector for thick-film electrodes (MicruX Technologies) was used. A 40 µL volume of PB, NaOH, or HNO_3_ solutions (depending on the study) was deposited on the electrochemical cell covering all the three electrodes (a 20 µL volume was used when SDS was added to remove PdNCs from the wells). Voltammograms (LSV) were recorded at room temperature from 0 to − 1.5 V with a scan rate of 50 mV s^−1^.

Other equipment employed in this work includes an EL × 800 absorbance microplate reader (Bio-Tek, USA), an ultrasonic bath (J.P. Selecta, Spain), a centrifuge (Gyrozen and Co., Rep. of Korea), a hotplate stirrer (Fisher Scientific, USA), a vortex mixer (Labbox Labware, Spain), and a stove (Memmert, Spain). The weighing was performed using a precision analytical balance (NewClassic MF, Mettler Toledo, Spain), and the pH was measured with a pHmeter (Crison micropH 200, Crison Instruments S.A., Spain).

### Human serum samples

Serum samples from five patients with IS and three with HS admitted to the Stroke Unit of Hospital Universitario Central de Asturias (HUCA, Oviedo, Spain) were analyzed in this work. All samples were collected within 48 h after symptomatology onset. Upon admission, a neurological examination was performed on all selected patients using the NIH Stroke Scale (NIHSS) together with a brain computed tomography scan. Additionally, four samples from CT donors (without any known pathology) were also analyzed to determine GFAP basal levels. In all cases, blood was collected in 5 mL Z Serum Sep Clot Activator tubes coated with microscopic silica particles, which activate the coagulation process (Vacuette, Madrid, Spain). Tubes were centrifuged at 1800 g for 18 min at 4 °C, and the supernatant (serum) was stored at − 80 °C until use. The procedures adhered to the tenets of the Declaration of Helsinki on biomedical research involving human subjects. Full ethical approval was obtained for the Clinical Research Ethics Committee at the Principality of Asturias (Oviedo, Spain) (Ref. ES330440003591).

## Methods

### Synthesis of PdNCs and labelling of anti-hGFAP

**Fig. 1:**
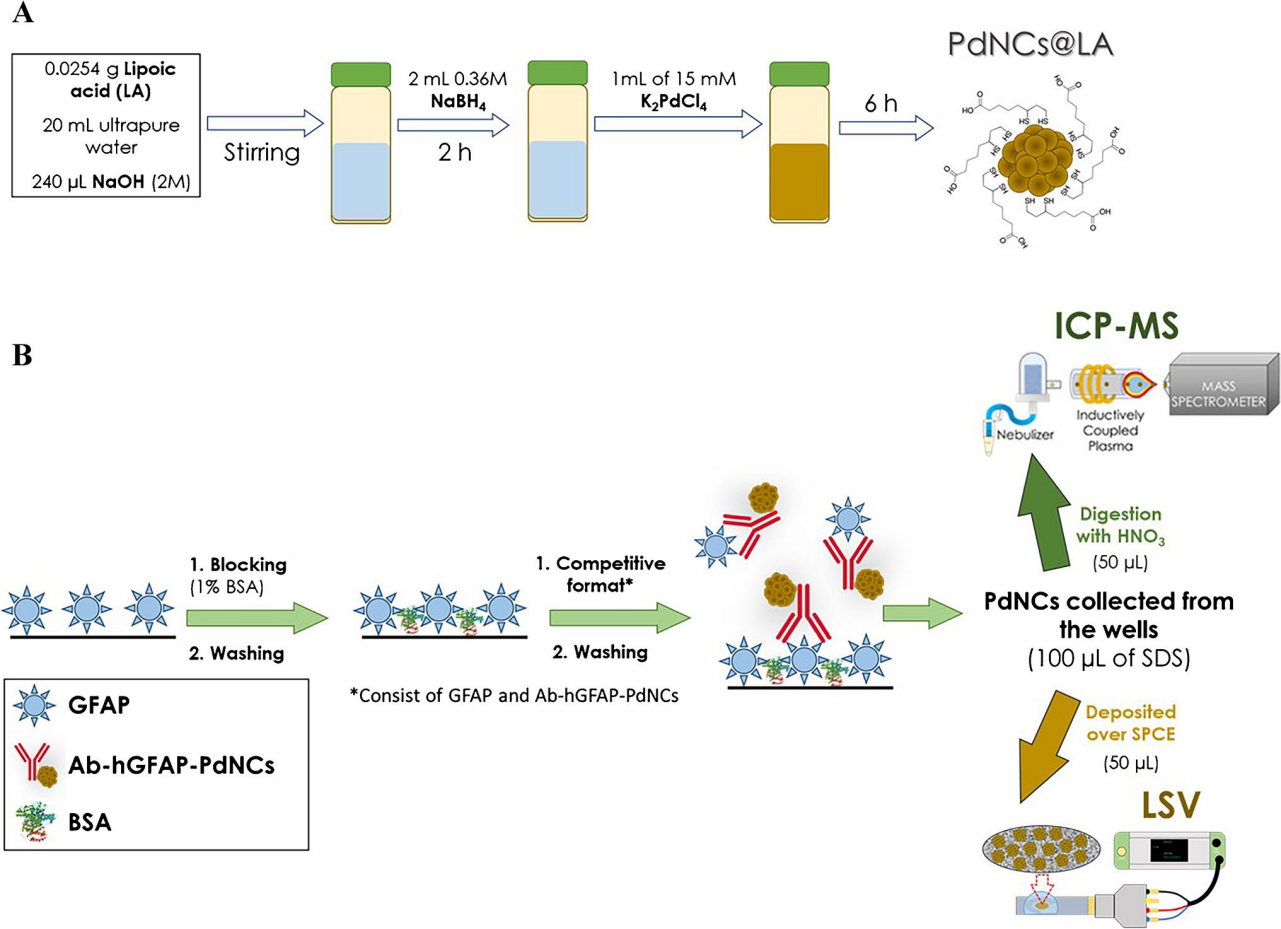
Schematic diagrams summarizing the proposed methodology, covering both the synthesis of PdNCs employed as labels and the immunoassay for the specific detection of GFAP by ICP-MS and LSV. **A** Schematic representation of the step-by-step experimental procedure employed to achieve stable and monodisperse PdNCs in aqueous media, and **B** scheme of the methodology for conducting the competitive immunoassay. This process begins with the coating of the plate with GFAP and concludes with the extraction of PdNCs from the wells using 8% SDS for subsequent detection. Both schematics are presented with the optimized experimental conditions

In a glass vial protected from sunlight, 0.0256 g of lipoic acid was dissolved in 20 mL of ultrapure water and 240 µL of 2 M NaOH. The vial was then placed in an ultrasonic bath for 5 min and was subsequently placed in a bath at a controlled temperature (9 °C, 25 °C, and 50 °C were investigated) being shaken vigorously for 5 min. Subsequently, 2 mL of NaBH_4_ (at concentrations of 0.16, 0.24, and 0.36 M) was added to the solution. After allowing the reaction to proceed for 2 h, 1 mL of 15 mM K_2_PdCl_4_ was added rapidly, and stirring was maintained for 6 h while continuously controlling the synthesis temperature to keep it constant. After the synthesis, PdNCs were purified by ultracentrifugation using 3 kDa membrane filters at 5500 rpm for 10 min. Finally, three additional washing steps with ultrapure water (5500 rpm for 10 min) were performed. The synthesis procedure for PdNCs is schematically outlined in Fig. 1A.

For the labelling of anti-hGFAP with PdNCs (Ab to PdNC immunoprobe), 100 µL of the anti-hGFAP (100 µg mL^−1^) together with the required amount of purified PdNCs (372 µL for the 1:5 molar ratio Ab to PdNCs) dissolved in 10 mM PB was added to an Eppendorf tube and vortexed. Subsequently, 12 μL of a solution containing EDC and NHS was added with a molar ratio Ab to EDC to NHS of 1:1500:1500. After 2 h at room temperature with constant stirring, purification was performed by ultrafiltration (100 kDa pore size Amicon filters) with a first cycle at 4000 rpm for 10 min and three washing steps with 10 mM PBS (4000 rpm for 10 min). The purified solution was stored in the fridge until use. For the competitive immunoassay for GFAP determination, the labelling of the Ab for the synthesis of the immunoprobe (anti-hGFAP to PdNCs) was performed on the same day as the competitive immunoassay.

### Immunoassay to study the optimal Ab to NC ratio in the immunoprobe

To investigate the optimal Ab to NC ratio in the immunoprobe, a microtiter plate was utilized. Each well was covered with 100 µL of GFAP used as standard (10 µg mL^−1^), except for the procedural blank where 10 mM PB solution was added, for 6 h at 37 °C. After removing the excess protein solution, free binding sites on the wells were blocked incubating with 1% BSA solution in PBS for 12 h at low temperature (3 °C in the fridge). Then, 200 μL of 10 mM PBS with 0.05% Tween 20 was used for washing (three cycles). Afterwards, 100 μL of the Ab to PdNC immunoprobe (1 μg mL^−1^) was added to the wells. Different Ab to PdNC molar ratios were investigated (in the range of 1:1 to 1:10). The mixture was incubated for 2 h at 37 °C. Then, the wells were washed (200 μL of 10 mM PBS with 0.05% Tween 20; three cycles), and 100 μL/well of the extraction solution (concentrated HNO_3_) was added and kept at room temperature for 30 min to ensure complete extraction of the PdNCs. After that, the extracted PdNCs were digested for 15 min in an ultrasonic bath, and the Pd concentration was determined by ICP-MS. Triplicates were prepared in all cases.

To investigate the optimal Ab to NC ratio in the immunoprobe, a microtiter plate was utilized. Each well was covered with 100 µL of GFAP used as standard (10 µg mL^−1^), except for the procedural blank where 10 mM PB solution was added, for 6 h at 37 °C. After removing the excess protein solution, free binding sites on the wells were blocked incubating with 1% BSA solution in PBS for 12 h at low temperature (3 °C in the fridge). Then, 200 μL of 10 mM PBS with 0.05% Tween 20 was used for washing (three cycles). Afterwards, 100 μL of the Ab to PdNC immunoprobe (1 μg mL^−1^) was added to the wells. Different Ab to PdNC molar ratios were investigated (in the range of 1:1 to 1:10). The mixture was incubated for 2 h at 37 °C. Then, the wells were washed (200 μL of 10 mM PBS with 0.05% Tween 20; three cycles), and 100 μL/well of the extraction solution (concentrated HNO3) was added and kept at room temperature for 30 min to ensure complete extraction of the PdNCs. After that, the extracted PdNCs were digested for 15 min in an ultrasonic bath, and the Pd concentration was determined by ICP-MS. Triplicates were prepared in all cases.

### Direct competitive immunoassays using PdNCs as a label

A competitive immunoassay was developed to determine GFAP in human serum samples. For such purpose, each well of a microtiter plate was covered with 100 µL of GFAP (5 µg mL^−1^) and incubated for 2 h at 37 °C. After removing the excess protein solution, free binding sites on the wells were blocked incubating with 1% BSA solution in PBS for 2 h at room temperature and then washed with 200 μL of 10 mM PBS with 0.05% Tween 20 (three cycles). Meanwhile, 50 μL of the specific PdNC immunoprobe (6 μg mL^−1^ of anti-hGFAP) was incubated in an Eppendorf tube with 50 μL of the protein standard (within the range of 5.32 aM to 532 pM of GFAP) or human serum sample and stirred for 15 min with a vortex. Afterwards, this mixture was added to the previously treated wells and incubated 2 h at 37 °C. Then, the wells were exhaustively washed (200 μL of 10 mM PBS with 0.05% Tween 20; four cycles), and 100 μL/well of the extraction solution (HNO_3_, SDS, or the mixture SDS–0.1 M mercaptoethanol: different reagents were investigated) was added to quantitatively extract the PdNCs (30 min at room temperature). When SDS (pure or mixed with mercaptoethanol) was used for the extraction, 100 µL of the desired SDS concentration was added to each well, and the plate was shaken at 700 rpm for 1 h at 37 °C. Three immunoassay triplicates were prepared in all cases, except for human serum samples that were analyzed by quadruplicate.

After extraction of PdNCs, the solutions were divided into two aliquots for detection by ICP-MS and LSV. GFAP was determined by ICP-MS measuring the Pd signal from the PdNC label, and by LSV taking as analytical signal the current intensity (owing to the catalytic activity of PdNCs over HER) at a fixed potential. For conventional nebulization ICP-MS analysis, 100 μL of concentrated HNO_3_ was added to one 50 μL aliquot to oxidize and dissolve Pd. Then, the tubes were introduced in an ultrasonic bath and maintained for 15 min. Finally, a 1:75 dilution (1150 μL final volume) of the samples was required to minimize the percentage of acid introduced into the ICP-MS (< 3% v/v). ICP-MS measurements were performed by external calibration with pure Pd standards (concentrations from 0.05 to 5 ng g^−1^) using Rh as the internal standard (isotopes ^105^Pd and ^103^Rh were measured, respectively). In the case of electrochemical measurements, the other aliquot, of 6 µL, was directly deposited on the working electrode, leaving them until the solution has dried. Finally, 40 μL of 0.5 M HNO_3_ was added for performing LSV measurements. It should be noted that each measurement was conducted using a new and disposable SPCE. The scheme of the step-by-step experimental procedure for conducting the competitive immunoassay is shown in Fig. 1B.

### Statistics

The results shown in the figures and tables are expressed as the mean value ± standard deviation (SD). The paired sample Student *t*-test at 95% confidence level was used to determine significant differences between data sets. Prior to this analysis, we conducted an *F*-test to assess the equality of variances between both populations. Significant differences are denoted as *p* < 0.05 (*), *p* < 0.01(**), and *p* < 0.005 (***).

Calibrations of the competitive immunoassays were performed using a 4-parameter logistic (PL), whose equation is shown in Eq. 1:

1 $$Y=d+ \frac{a-d}{1+{\left(\frac{X}{c}\right)}^{b}}$$

where “*a*” and “*d*” are respectively the points of highest and lowest signal response, “*b*” denotes the slope of the curve at the inflection point, and “*c*” corresponds to the inflection point.

## Results and discussion

### Synthesis and characterization of PdNCs

The PdNC synthesis process was carefully optimized to ensure the production of stable and monodisperse PdNCs with catalytic properties over HER. PdNCs were synthesized following the procedure described in the “Experimental” section using NaBH_4_ as a reducing agent and lipoic acid as a small thiolated stabilizing agent. Several parameters, including the temperature (9, 25, and 50 °C) as well as the concentration of the reducing agent (0.16, 0.24, and 0.36 M NaBH_4_ solution) were evaluated for the synthesis. PdNCs were first characterized by spectrophotometry. As an example, Fig. S1 illustrates the absorbance spectra obtained for the PdNC suspension (synthesized at 50 °C with 0.36 M NaBH_4_) where two peaks at 367 and 279 nm can be observed. Such maxima did not appear neither in the solution of the salt precursor nor in the blank of the synthesis. EDX measurements confirmed the presence of Pd from NCs and S from the thiolated stabilizing agent (Fig. S2). Similar absorbance spectra were obtained when comparing this spectrum with those obtained with 0.24 M NaBH_4_ or at 25 °C (Fig. S3).

**Fig. 2:**
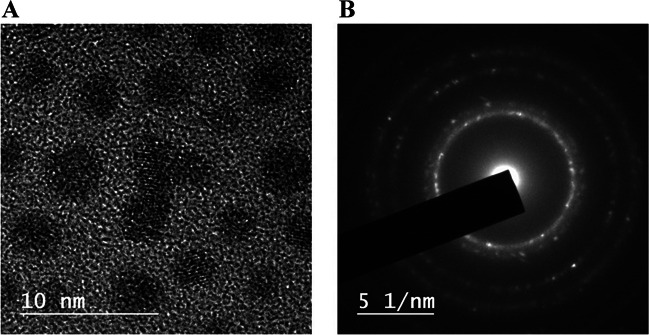
Structural characterization of PdNCs synthesized at the selected experimental conditions (50 °C and 0.36 M NaBH_4_). **A** HR-TEM image of the purified PdNCs, and **B** selected area electron diffraction pattern of PdNCs determined by HR-TEM

The formation of the PdNCs was confirmed by HR-TEM (Fig. 2A). PdNC diameter was determined by measuring PdNCs observed in HR-TEM images. PdNCs have an average diameter of 2.49 ± 0.02 nm (determined for *n* = 300, confidence interval 99%, standard normal distribution) with a round shape. PdNCs showed a face-centered cubic crystal lattice, which proved measuring the d_spacing_ values from the selected area electron diffraction (SAED) pattern shown in Fig. 2B. Compared to another synthesis strategy recently proposed for PdNCs [23], the use of lipoic acid as stabilizing agent in comparison to DL-6,8-thioctic acid (DHLA) allowed obtaining larger PdNCs with a lower polydispersity (1.69 ± 0.06 nm using DHLA). A larger size should provide a higher sensitivity by ICP-MS detection (higher number of Pd atoms per NC). In addition, the dispersion associated with the PdNC diameter generates a variability in the number of metal atoms per nanostructure (the standard deviation in the PdNC diameter is directly related to the number of Pd atoms per NC), limiting the possibility of obtaining accurate and precise quantification of the target protein by elemental ICP-MS. Therefore, it could be expected that comparatively bigger and less polydispersed PdNCs, synthesized with lipoic acid, would offer better analytical performance for the determination of biomolecules by ICP-MS detection. These results suggest that the selected synthesis conditions were effective in producing PdNCs with consistent size and stability. This will facilitate their use as antibody tags, as the ligand and metal nucleus will establish a robust bond, thereby enhancing the accuracy of both ICP-MS and LSV measurements. Their low polydispersity will reduce the error associated with fluctuations in the size and hence the properties of the PdNCs.

Purified PdNC suspensions were analyzed by conventional nebulization ICP-MS to determine Pd concentration. Table 1 collects the Pd concentrations obtained by ICP-MS in the PdNC suspensions synthesized at different temperatures and different reducing agent concentrations. Table 1 also includes the synthesis yield obtained, considering the Pd concentrations determined in the solutions of the Pd salt precursor and in the purified PdNC suspensions. As can be observed, a similar synthesis yield (average value about 54%) was obtained at a low temperature (9 °C), independently of the concentration of the reducing agent (0.16–0.36 M). However, improvements in the synthesis yield were observed at higher temperatures (25 and 50 °C) by increasing the concentration of NaBH_4_. Thus, a synthesis yield of 60% was observed using 50 °C and 0.36 M NaBH_4_, and such experimental conditions were selected for the PdNC synthesis.

**Table 1:** Pd concentrations determined by ICP-MS in the purified PdNC suspensions synthesized using different temperatures and reducing agent concentrations. Uncertainties represent the standard deviations of the mean of three PdNC suspensions. The Table also lists the synthesis yield, expressed as percentage

Temperature (°C)	[NaBH_4_] (M)	[Pd] (µg mL^−1^)	PdNC synthesis yield (%)
9	0.16	36 ± 1.9	52
	0.24	37 ± 2.2	53
	0.36	39 ± 1.0	56
25	0.16	25.2 ± 0.6	36
	0.24	35 ± 1.2	50
	0.36	41 ± 1.0	59
50	0.16	27 ± 1.1	39
	0.24	33.9 ± 0.7	48
	0.36	41.6 ± 0.8	60

The concentration of Pd at the optimized synthesis conditions was determined by ICP-MS in three replicates of the purified PdNC suspensions after their acid digestion. Experimental results showed a Pd concentration of 0.405 ± 0.008 mM. Calculations to obtain the number of Pd atoms per NC are collected in the ESM. The ratio between the elemental concentration of Pd in solution and the number of Pd atoms per NC (average value of 550 atoms of Pd) gives the concentration of PdNCs in the synthesis solution, which resulted to be 7.4⋅10^−7^ mol NCs L^−1^.

### Evaluation of PdNC catalytic activity on HER

Cyclic voltammetry (CV) was selected to check the catalytic activity of the synthesized PdNCs and evaluate the possible effect of the pH. Thus, PdNC suspensions were diluted in different media in such a way that basic (0.1 M NaOH), neutral (0.1 M PB pH 7.6), and acidic (0.1 M HNO_3_) conditions were tested. Fig. S4 shows the voltammograms obtained by CV (sweeping the potential between − 1.5 and + 1.5 V) for the procedural blanks and the purified PdNC suspensions (with a 1:25 dilution in the different media). It can be seen that there was not any electrochemical process of interest in the basic medium (Fig. S4A). In the case of neutral and acidic media, PdNCs exhibited catalytic activity on HER (Fig. S4B and S4C, respectively), being such activity significantly higher in the acidic medium. Thus, the acidic medium allows obtaining better sensitivity compared to the neutral and, therefore, it was selected for further electrochemical experiments. Furthermore, LSV was selected for further analyses recording voltammograms between 0 and − 1.5 V since the backward scan in CV did not provide any additional analytical information.

**Fig. 3:**
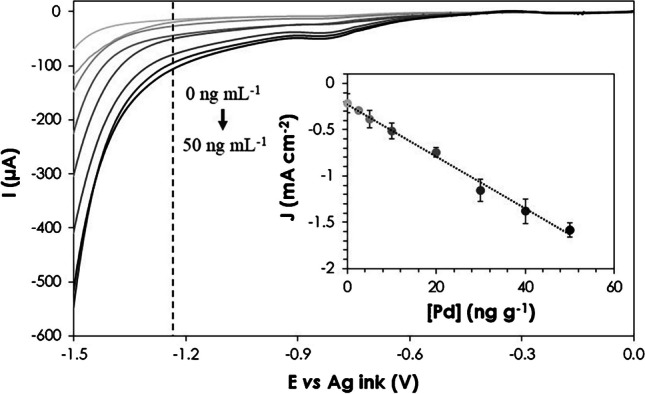
Voltammograms obtained by LSV for the study of PdNC catalytic activity on HER. The inset shows the current density measured at a constant potential of − 1.25 V after deposition of 6 mL of purified PdNC solution (in the range of 0–50 ng mL^−1^). Concentration increases from light (0 ng mL^−1^) to dark (50 ng mL^−1^) gray color. Uncertainties represent the standard deviations of the mean of three measurements

The influence of the PdNC concentration on the catalytic activity on HER was subsequently investigated by LSV, measuring the current intensity at which a given potential is achieved. Figure 3 depicts the calibration graph obtained by depositing 6 µL of the purified PdNC suspension (in a concentration range comprised between 0 and 50 ng mL^−1^ of Pd) over the working electrode of a SPCE and letting dry for 1 h at 37 °C. The calibration was performed by adding onto such PdNC-modified electrode 40 µL of 0.5 M HNO_3_ (to ensure acidic medium) and measuring the intensity achieved at a constant potential of − 1.25 V. As can be seen, there was a good linear relationship (*R*^2^ = 0.991) between the current density (J, in mA cm^−2^) and the Pd concentration, with a sensitivity of − 0.035 mA mL ng^−1^ cm^−2^. It is noteworthy that the measurements exhibited an average relative standard deviation (RSD) of 6%. The optimal potential selected was − 1.25 V, as calibration curves at − 1.20 V and − 1.30 V exhibited poorer linear fits (*R*^2^ = 0.986 and *R*^2^ = 0.981, respectively). While there was a slight increase in sensitivity when using − 1.30 V (− 0.042 mA mL ng^−1^ cm^−2^), the precision of the measurements decreased, with a RSD value of 8%. Consequently, − 1.25 V was selected as the optimum potential for future measurements.

Therefore, the catalytic activity over HER exhibited by PdNCs is directly proportional to the concentration of PdNCs, and hence, it would be possible to use such PdNC catalytic activity for bioanalytical purposes.

### Optimization of the competitive immunoassay

**Fig. 4:**
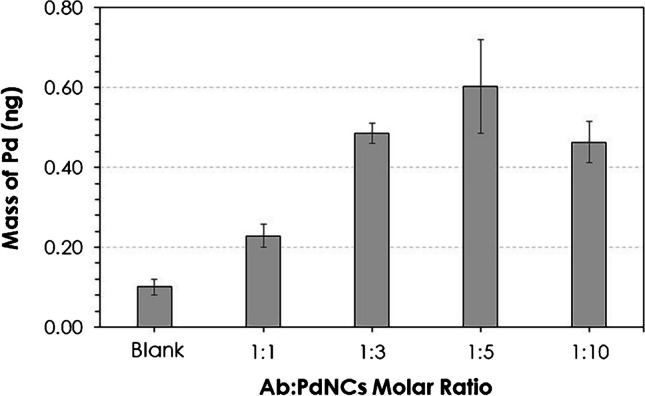
Mass of Pd (ng of Pd collected from the ELISA well plates) measured by ICP-MS for different Ab to PdNC molar ratios employed in the reaction solution for anti-hGFAP labelling. Uncertainties represent the standard deviations of the mean of three independent measurements

Once PdNCs were characterized, they were bioconjugated to label the anti-hGFAP by following the carbodiimide method, following the procedure described in the “Experimental” section. The recognition capabilities of the immunoprobe (Ab to PdNCs) were studied as a function of the Ab to PdNC molar ratio added to the Ab labelling reaction. For such purpose, the immunoassay was carried out investigating different Ab to PdNC molar ratios (1:1, 1:3, 1:5, and 1:10), and Pd was determined by ICP-MS. Figure 4 presents the mass of Pd calculated by ICP-MS depending on the Ab to PdNC molar ratio employed in the labelling solution. A procedural blank (PB solution without GFAP) was employed to evaluate nonspecific interactions of the immunoprobe. As can be seen, the mass of Pd increased from an average value of 0.23 ng of Pd for the 1:1 molar ratio up to 0.60 ng of Pd for the 1:5 molar ratio, indicating that the amplification provided by PdNC label increased when raising the Ab to PdNC molar ratio from 1:1 to 1:5 (i.e., a higher number of Pd atoms are available per Ab molecule). However, it was observed that the Ab functionality decreased when the Ab to PdNC molar ratio increased up to 1:10 (0.46 ng of Pd). This could be attributed to the fact that the PdNCs could block the recognition sites of the Ab molecules [22]. Thus, a 1:5 molar ratio was selected as the optimum since a higher signal is achieved by ICP-MS detection.

**Fig. 5:**
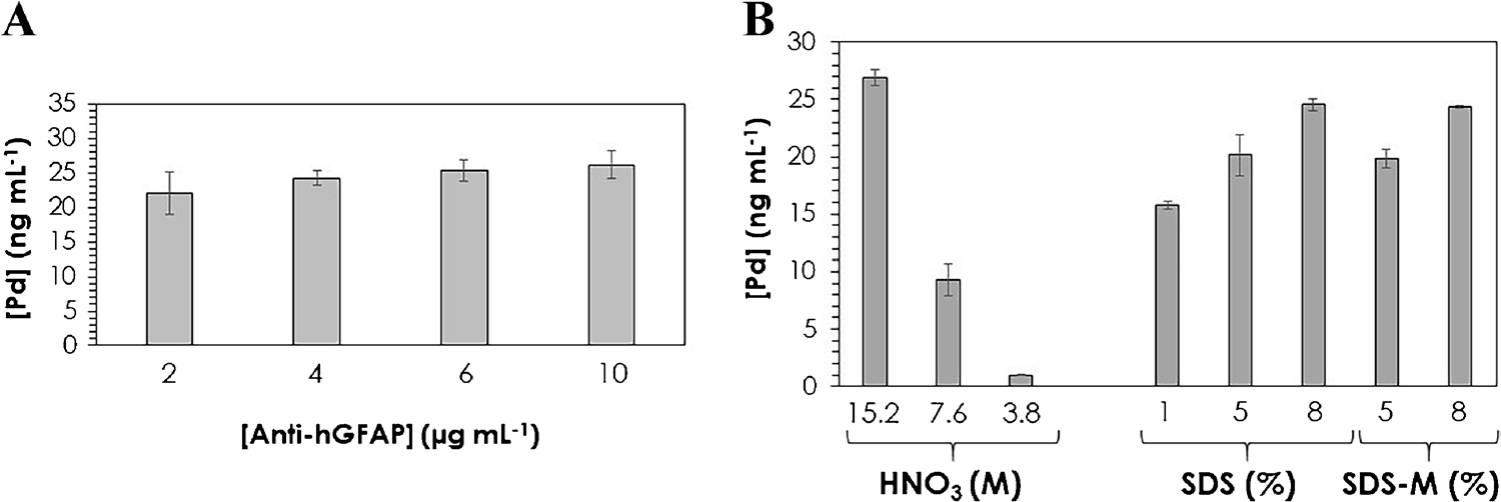
Concentration of Pd determined by ICP-MS in the samples extracted from the wells after the competitive immunoassay for GFAP determination using PdNC immunoprobe. **A** Study of anti-hGFAP concentration, and **B** evaluation of different media for the extraction of PdNCs (M = 0.1 M b-mercaptoethanol). Uncertainties represent the standard deviations of the mean of three independent measurements

Taking into account the low protein concentration expected in CT and IS human serum samples [7], optimization of the competitive immunoassay for GFAP determination was carried out in terms of the concentration of the anti-hGFAP and the reagent employed for the extraction of PdNCs from the wells. Figure 5 depicts the optimizations carried out to select the Ab concentration and the extraction reagent by measuring Pd concentration by ICP-MS. The concentration of the anti-hGFAP in the immunoprobe used in the competitive step was studied in the range between 2 and 10 μg mL^−1^ (Fig. 5A). A concentration of Ab of 6 µg mL^−1^ was selected as optimum because the Pd concentration was similar to that obtained when using 10 µg mL^−1^, and thus, reagent is saved without decreasing the sensitivity. Lower concentrations exhibited lower Pd levels and higher uncertainty.

Concerning the reagent used for the extraction, media with different compositions and concentrations were evaluated to extract PdNCs from the wells: HNO_3_, SDS, and the mixture SDS–mercaptoethanol (Fig. 5B). Concentrated HNO_3_, 5% SDS–0.1 M mercaptoethanol, 8% SDS–0.1 M mercaptoethanol, 5 and 8% SDS allowed an efficient extraction of PdNCs from the wells, whereas lower concentrations of HNO_3_ or SDS did not allow full recovery. The main limitation of employing concentrated HNO_3_ is the subsequent dilution required for analysis both by ICP-MS and LSV: solutions must not exceed a 3% of acid content for ICP-MS and a high acidic medium could be harmful to the electrodes. An 8% SDS solution was selected as the extraction reagent for further experiments, because lower uncertainty was observed than when using 5% SDS (the SDS–mercaptoethanol mixture also showed quantitative recovery for Pd, but, since mercaptoethanol is a chemical product hazardous to health, it was finally discarded). The influence of SDS (surfactant) on ICP-MS and LSV analyses was evaluated by comparing calibration curves performed with and without SDS. Experimental results concluded that no effects were observed by ICP-MS, while a slight improvement in the current intensity was found using SDS for LSV measurements. This fact agrees with previous studies reported for another type of nanoparticles; higher signals could be due to the fact that small H_2_ bubbles that are formed on the electrode are more easily desorbed [25].

Finally, the real number of PdNCs per Ab (i.e., the immunoprobe stoichiometry) was estimated. For such purpose, a competitive immunoassay with GFAP (described in the “Experimental” section) was carried out and the Pd concentration in the bound immunoprobe solutions (Ab to PdNCs) containing a known amount of anti-hGFAP was calculated by ICP-MS. Fig. S5 collects the relationship obtained between the concentrations of Pd and GFAP (both in pmoles). The number of Pd atoms per GFAP molecule in solution for the 1:5 Ab to PdNCs was found to be 1647 on average, corresponding to approximately three PdNCs per Ab (550 Pd atoms per NC).

### Application of bimodal determination of GFAP to human serum samples: ICP-MS and LSV

**Fig. 6:**
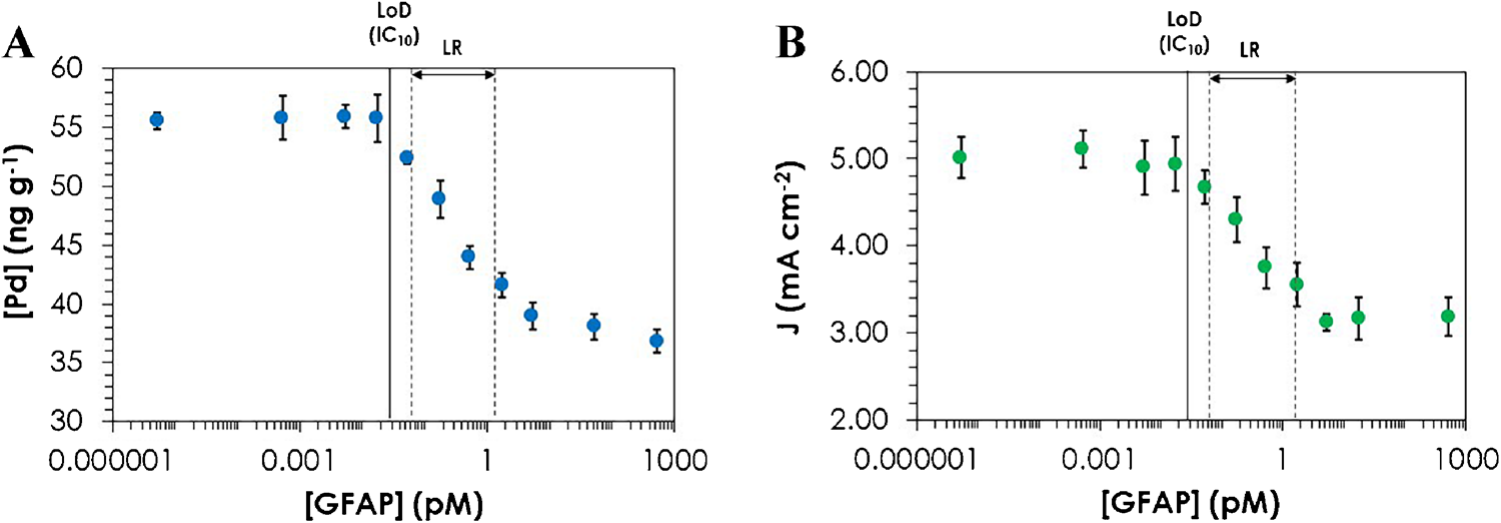
Calibration curves obtained with the competitive immunoassay for GFAP determination using PdNCs as label by elemental MS and electrochemical detection. **A** Calibration graph obtained by ICP-MS, and **B** calibration graph obtained by LSV (current intensity measured at − 1.25 V). Uncertainties represent the standard deviations of the mean of three independent measurements for ICP-MS analyses and four independent measurements for LSV analyses. LoD, limit of detection (IC_10_); LR, linear range

The applicability of the PdNCs as a label in immunoassays for further ICP-MS and LSV detection was evaluated by quantifying GFAP in human serum samples. Figure 6 shows the calibration graphs (within the range of 5.32 aM to 532 pM of GFAP) by ICP-MS and LSV. In the case of ICP-MS (Fig. 6A), a calibration plot was constructed by representing the concentration of Pd in the wells as a function of the concentration of the GFAP standards. The higher the concentration of GFAP, the lower the Pd concentration (competitive immunoassay). The calibration plot was fitted with a 4-PL curve with MyAssays Ltd., resulting in a *R*^2^ value of 0.993 (Eq. 2). We also extracted different inhibitory concentration (IC) values from the graph. The limit of detection (LoD), corresponding to IC_10_, was 0.03 pM of GFAP, while the linear range (IC_20_–IC_80_) extended from 0.06 up to 1.5 pM of GFAP. For LSV analyses, the current intensity due to catalyzed HER was measured at a constant potential (− 1.25 V) for the different GFAP concentrations (Fig. 6B). Similarly to ICP-MS analyses, it was adjusted with a 4-PL curve, resulting in Eq. 3 with a *R*^2^ value of 0.992.

2 $$j \left(\frac{mA}{{cm}^{2}}\right)=36.6+ \frac{55.8-36.6}{1+{\left(\frac{\left[GFAP \right] (pM)}{0.28}\right)}^{0.65}}$$

3 $$j \left(\frac{mA}{{cm}^{2}}\right)=3.13+ \frac{5.04-3.13}{1+{\left(\frac{\left[GFAP\right] (pM)}{0.28}\right)}^{0.91}}$$

The response interval for LSV detection was similar to that obtained with ICP-MS (between 0.06 and 1.3 pM of GFAP), and the same LoD was found (0.03 pM of GFAP). However, the uncertainty associated with measurements was significantly higher for LSV measurements.

There are several ways to determine the LoD in competitive immunoassays when a 4-PL fit is performed. One of the most widespread is to apply the IC_10_ method above indicated [26, 27], but this does not consider the standard deviation of the calibration measurements. One of the most robust ways to calculate the LoD is using the error profile method [28], where the lowest detectable concentration is calculated by interpolating the intersection of the lower asymptote for 95% confidence in the 4-PL fit performed with the average values [29]. To do this, first, it is needed to adjust 4-PL with the calibration points. Then the calibration must be redrawn, using as data the lower values obtained for a 95% confidence interval instead of the average values. Finally, the resulting parameter “*a*” is introduced in the 4-PL equation obtained for the average values to obtain the minimum detectable concentration, i.e., the LoD. When measuring by ICP-MS, the LoD calculated by the error profile method was again 0.03 pM, while 0.11 pM was obtained for LSV measurements. In this way, the differences between the two ways of calculating the LoD can be appreciated: LoD values are the same for both measurement techniques by the IC_10_ method; however, when applying the error profile method, the LoD is lower by ICP-MS, due to the better precision of the measurements. In any case, with both detection methods and following both modes of LoD calculation, the values obtained are much lower than the 0.31 pM LoD provided by the commercial kit.

The GFAP concentration in human serum samples from five patients with IS, three with HS, and four donors without known neurocerebral pathology (controls) was determined using the PdNC-based competitive immunoassay. Four independent measurements were performed in ICP-MS and six for LSV for the analysis of human serum. As reported in the “Experimental” section, after performing the competitive immunoassay the well content was divided into two aliquots that were subsequently measured by ICP-MS and LSV. Additionally, the results obtained with the PdNC-based immunoassay were compared with those obtained by a commercial ELISA kit. Table 2 collects the GFAP concentrations in human serum samples obtained using the ELISA kit with the PdNC immunoprobe by both ICP-MS and LSV. The experimental values obtained for GFAP in human serum were in the same range as concentrations previously reported by other authors [10]. Note that differences found in the GFAP level between published works can be attributed to the time of sampling collection after the stroke episode (within 48 h in our case).

**Table 2:** Experimental results obtained for the determination of GFAP in human serum samples from HS, IS and CT donors using PdNC immunoprobe (anti-hGFAP to PdNCs) by ICP-MS and LSV. The Table also collects the values obtained for GFAP concentration determined by a commercial ELISA kit. Uncertainties represent the standard deviations of the mean of three independent immunoassays for ELISA kit, four independent measurements for ICP-MS and six independent measurements for LSV

s		[GFAP] (pM)		
		ICP-MS	LSV	ELISA kit
IS	IS-1	5 ± 1	4 ± 2	5.1 ± 0.7
	IS-2	10 ± 1.2	16 ± 7	11.2 ± 0.8
	IS-3	0.09 ± 0.02	< LoD	< LoD
	IS-4	13 ± 2	19 ± 12	17 ± 3
	IS-5	33 ± 5	33 ± 8	35 ± 7
HS	HS-1	1200 ± 220	1500 ± 640	1290 ± 90
	HS-2	210 ± 60	400 ± 110	300 ± 120
	HS-3	2200 ± 300	2000 ± 1120	2190 ± 70
CT	CT-1	< LoD	< LoD	< LoD
	CT-2	0.07 ± 0.04	< LoD	< LoD
	CT-3	0.03 ± 0.02	< LoD	< LoD
	CT-4	0.05 ± 0.03	< LoD	< LoD

Regarding the accuracy, no significant differences were found applying a paired sample Student *t*-test at 95% confidence to the experimental results obtained with the PdNC-based immunoassay and the ELISA kit. The *p*-values were as follows: 0.9255 and 0.9779 for ICP-MS results compared to ELISA (IS and HS, respectively) and 0.8781 and 0.9604 for LSV results compared to ELISA (IS and HS, respectively). It should be highlighted that GFAP concentration in CT samples could be only detected by ICP-MS, being the low protein level below LoD both for LSV and ELISA kit. It was also the case for the IS sample with the lowest GFAP concentration (IS-3): only by using PdNC-based immunoassay and ICP-MS detection; it was possible to determine GFAP level in the human serum sample. Concerning the precision associated with GFAP determination with the different analytical methods, similar repeatability values were found for ICP-MS and the ELISA kit for IS and HS samples. However, as can be seen in Table 2, higher uncertainty was associated with LSV measurements. Note that the uncertainty associated with GFAP concentration in CT samples was significantly higher in comparison to IS and HS samples. This fact can be attributed to the low protein level for CT donors.

**Fig. 7:**
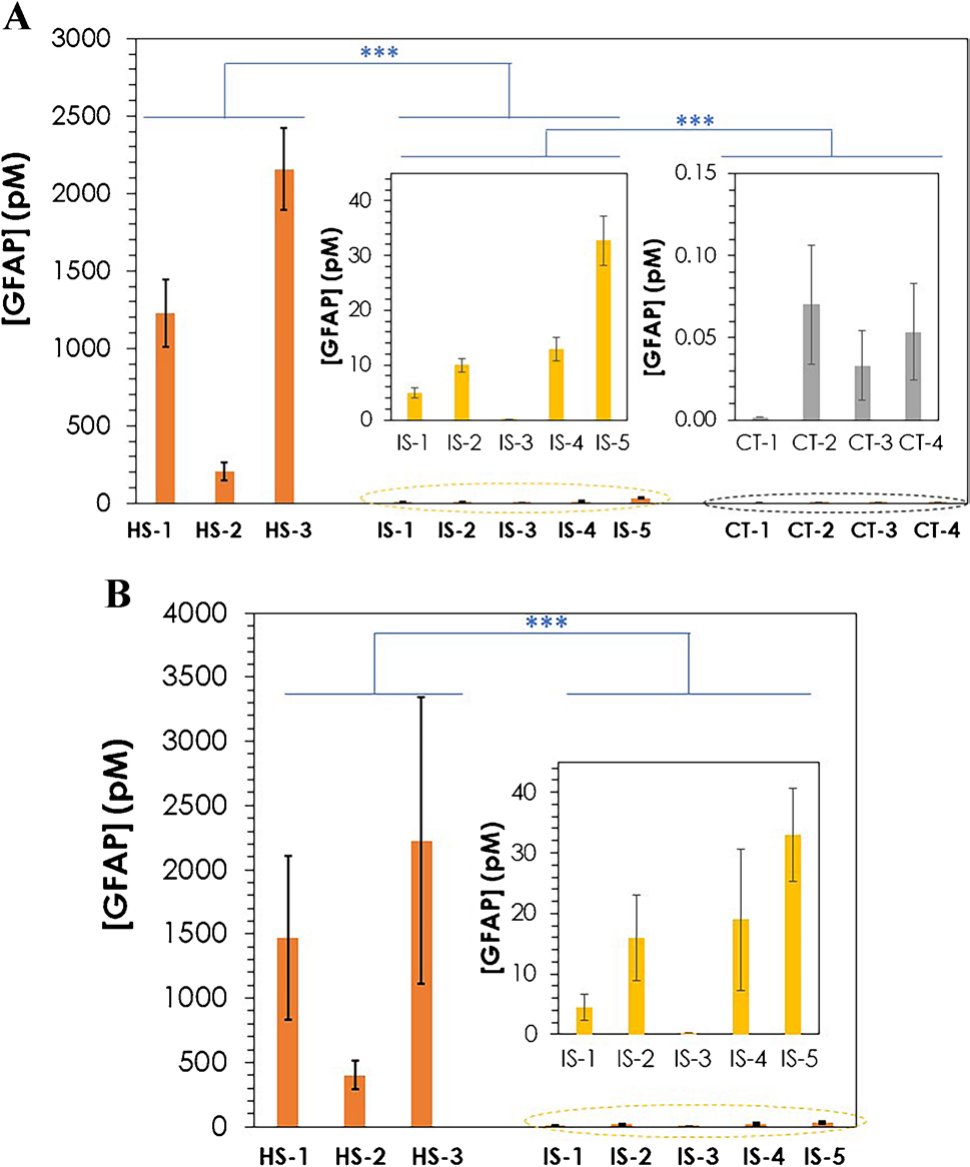
GFAP concentrations determined by ICP-MS in HS, IS and CT human serum samples by ICP-MS (**A**) and LSV (**B**), after a competitive immunoassay using PdNC immunoprobe. For a better data evaluation, the inserts depict the concentrations obtained for IS and CT samples with a different scale. Uncertainties represent in all cases the standard deviations of the mean of four independent measurements for ICP-MS analysis and six independent measurements for LSV. ***p-value < 0.001 (*t*-test at 95% confidence assuming unequal variances). In particular, *p*-value < 2.2·10^−7^ and < 7.1·10^−7^ when comparing HS with IS (ICP-MS and LSV, respectively) and *p*-value < 4.4·10^−6^ when comparing IS with CT (ICP-MS)

Fig. 7 shows a plot with the GFAP concentration determined by ICP-MS (Fig. 7A) and LSV (Fig. 7B) in the human serum samples. As can be graphically observed, GFAP was highly overexpressed in HS samples compared to IS and CT. Although a significant variation in GFAP concentration was observed between individuals within the same group (HS, IS, or CT), a higher GFAP concentration was found for IS patients compared to CT donors. A *t*-test at 95% confidence assuming unequal variances was applied to the values obtained for IS and HS patients as well as CT donors. Statistical differences in GFAP concentration were found by comparing HS and IS samples (both by ICP-MS and LSV), as well as for IS versus CT samples (ICP-MS measurements), showing the capability of the proposed approaches for discriminating between groups.

Finally, the proposed methodology was compared with other previously reported methods for determining GFAP. Table 3 provides a summary of analytical strategies employed for GFAP detection, including the reported LoDs, as well as two comparative columns detailing the estimated analysis cost and the potential for adaptation to POC analysis. As can be seen, the PdNC-based immunoassay using both LSV and ICP-MS detection demonstrates outstanding LoDs, surpassing commercial immunoassays and some other strategies found in the literature (e.g., immunosorbent assay with Carbon Dots or electrochemiluminometric immunoassay) [12, 30]. While our LoDs fall within the range reported for an Au-based biosensor [31], they are worse than those achieved with immunoassays employing single molecule analysis (SIMOA) detection [15]. However, it should be noted that in these last two cases, the instrumentation is more complex than LSV, involving techniques like electrochemical impedance spectroscopy or the use of a specific fluorescence microscope for SIMOA.

**Table 3:** Comparison of the LoDs, cost and adaptability for use in the development of POC devices, for the determination of GFAP in serum following different strategies

Analytical technique	Reference	LoD (pg mL^−1^)	Low-cost	Easy adaptation to POC analysis
Competitive immunoassay using PdNCs (LSV detection)	This work	5.4	+ +	+ +
Competitive immunoassay using PdNCs (ICP-MS detection)	This work	1.5	−	−
Commercial ELISA kit	This work	15	+	+
Electrochemiluminometric immunoassay	[12]	30	+ +	+ +
Immunosorbent assay with carbon dots (CLAISA)	[30]	25	+	±
Anti-GFAP/Au-based biosensor (EIS)	[31]	1	+	+
Immunoassay with SIMOA detection	[15]	0.21	±	+

Code: (+ +) excellent, ( +) good, ( ±) acceptable, ( −) poor

Table 3 also presents other productivity-related aspects, such as the cost of the analysis and the possibility of decentralization. Despite the excellent performance offered by ICP-MS, this methodology requires relatively expensive instrumentation. The same applies to single-molecule array methods like SIMOA. The immunosorbent assay with Carbon Dots [30] necessitates a spectrofluorimeter, which remains a benchtop equipment. In this line, the stability of fluorophores and adaptation to portable image acquisition and processing techniques are ongoing challenges. On the other hand, although electrochemiluminometric methodology [12] uses a small and not particularly expensive equipment (due to the electrical nature of the measurement and the simplicity of the electronics required), the LoD provided is poorer than with our methodology. Finally, although commercial ELISA immunoassays typically use relatively affordable plate readers, decentralization is challenging as the assay is typically configured for a standard number of wells and is commonly conducted in centralized settings when a large number of samples need to be processed.

Regarding the methodologies developed in this work, when sensitive and reproducible measurements are needed, the recommendation is to employ ICP-MS methodologies. However, the electrochemical measurements used to determine catalytic activity (and therefore, GFAP concentration) are very easy to perform, since they only require the recording of a LSV curve. Therefore, when cost-effectiveness and decentralization become relevant, electrochemical detection should be considered. It is important to note that the PdNC-based immunoassay with LSV detection requires further refinement, especially in terms of precision, to make it suitable for analysis in the clinical field. One promising approach is to perform the immunoassay directly on disposable miniaturized electrochemical cells, which could potentially enhance analytical features such as precision (by eliminating critical steps like PdNC deposition) and reduce analysis time [32]. Considering that nowadays there is a wide catalogue of miniaturized commercial potentiostats, it could be easy to achieve a decentralized biosensor-based methodology using metal NCs as labels. For the methodology presented here, this would require the integration of the immunoassay with components, such as screen-printed electrodes [32, 33].

## Conclusion

Monodisperse PdNCs were synthesized and evaluated as label for the determination of a specific protein. PdNCs present catalytic activity on HER reaction that can be electroanalytically useful and also high amplification can be expected by ICP-MS detection due to the high number of Pd atoms per immunoprobe. Thus, the same assay for a target protein determination can be bimodally detected by elemental MS and electrochemical detection. GFAP detection in CT, IS, and HS human serum samples was selected as a model to check the analytical capabilities of both detection approaches. Results showed that both detection modes provide better LoDs than the ELISA commercial kit. Although the LoD obtained by ICP-MS was lower than by LSV due to the higher precision of the ICP-MS measurements, both techniques allow differential diagnosis between HS and IS.

ICP-MS and LSV are two extremely different techniques: while the electrochemical methodology could be developed for on-site analyses and screening purposes, ICP-MS requires bulky and more expensive laboratory instrumentation. However, ICP-MS is a multi-elemental technique, thus meaning that multiple target proteins can be analyzed simultaneously [19] provided that different elemental labels are used for each immunoprobe. In this way, the application of monodisperse PdNCs increases the palette of highly sensitive labels for ICP-MS detection. On the other hand, multi-detection in electrochemistry should be approached by developing immunosensors and using spatial separation strategies.

The low LoD demonstrated by the ICP-MS methodology could be very useful for the determination of GFAP in other types of samples where the concentration is not as high as in serum, such as nasal exudate. The concentration of GFAP in serum has recently been found to correlate with its concentration in nasal exudate [34]. We consider this biological sample as particularly valuable for decentralized analysis due to its ease of non-invasive and rapid collection, eliminating the need for specialized personnel or extensive sample processing.

Finally, results point toward the future use of PdNCs as a label in other immunoprobes for the determination of specific proteins requiring very low LoDs as well as the development of electrochemically decentralized methodologies that would allow its use in peripheral hospitals or health centers not having centralized equipment.

### Supplementary Information

Below is the link to the electronic supplementary material.

Supplementary file1 (DOCX 393 KB)

## Data Availability

The data that support the findings of this study are available under requestment.
