# Supplementary material for: Palladium nanoclusters as a label to determine GFAP in human serum from donors with stroke by bimodal detection: inductively coupled plasma-mass spectrometry and linear sweep voltammetry
Source: Mikrochim Acta. 2023 Nov 30;190(12):493. doi: 10.1007/s00604-023-06059-5 (PMC10689531; doi:10.1007/s00604-023-06059-5)
Supplement: Supplementary file 1 — Supplementary file1 (DOCX 393 KB) [file 604_2023_6059_MOESM1_ESM.docx]

Electronic Supporting Material on the Microchimica

Acta publication entitled

**Palladium Nanoclusters as Label to Determine GFAP in Human Serum from Donors with Stroke by Bimodal Detection: Inductively Coupled Plasma-Mass Spectrometry and Linear Sweep Voltammetry**

Alejandro Rodríguez-Penedo,^1^ Estefanía Costa-Rama,^1^ Beatriz Fernández,^1*^ Carmen García-Cabo,^2^ Lorena Benavente,^2^ Sergio Calleja,^2^ María Teresa Fernández-Abedul,^1*^ Rosario Pereiro^1^

^1^Department of Physical and Analytical Chemistry, University of Oviedo, Julian Clavería 8, 33006 Oviedo, Spain

^2^Department of Neurology, Hospital Universitario Central de Asturias (HUCA), Oviedo, Spain

*Corresponding authors email addresses:

[fernandezbeatriz@uniovi.es](mailto:fernandezbeatriz@uniovi.es) & [mtfernandeza@uniovi.es](mailto:mtfernandeza@uniovi.es)

**DESCRIPTION OF THE ELECTRONIC SUPPLEMENTARY MATERIAL**

ESM contains details about the Experimental and Results and Discussion sections. First, one Table with the experimental conditions employed for ICP-MS is depicts. Additionally, calculations to obtain the number of Pd atoms per NC are collected. Five Figures related to PdNCs characterization as well as LSV and ICP-MS measurements are also included in ESM.

**EXPERIMENTAL**

**Instrumentation**

**Table S1** depicts the experimental parameters optimized for ICP-MS analysis.

**Table S1.** Operating conditions for ICP-MS measurements.

| **Parameter** | **Values** |
| --- | --- |
| **ICP RF power (W)** | 1550 |
| **Plasma gas flow (L min^-1^)** | 15 |
| **Make up gas flow (L min^-1^)** | 0.10 |
| **Nebulizer gas flow (L min^-1^)** | 1.07 |
| **Isotopes (m/z)** | ^103^Rh,^105^Pd,^106^Pd |
| **Dwell time (ms)** | 500 |

**RESULTS AND DISCUSSION**

**Synthesis and Characterization of PdNCs**

Figures S1, S2 and S3 collect the experimental results obtained by spectrophotometry and EDX measurements for the PdNCs suspensions during the optimization of the synthesis process.


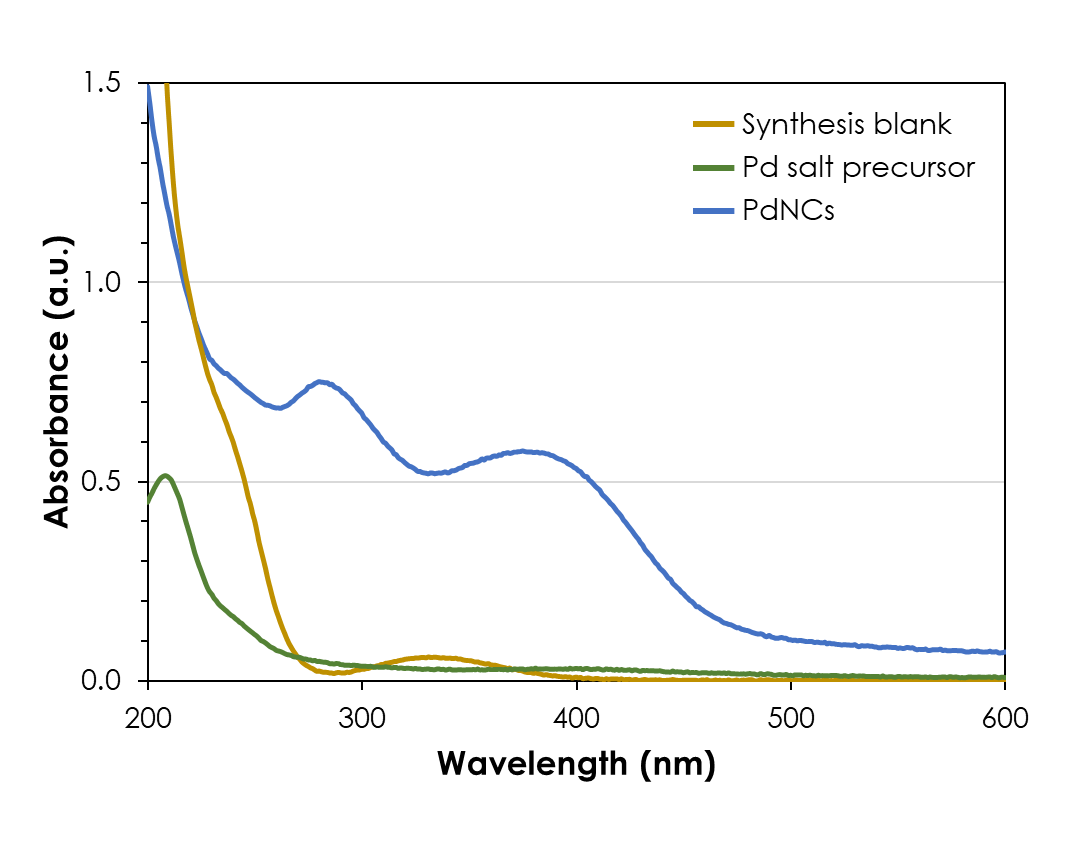


**Figure S1**. Absorbance spectra obtained for the blank of the synthesis, the solution of the Pd salt precursor (K_2_PdCl_4_) and the purified PdNCs solution (synthesized at 50ºC with 0.36 M NaBH_4_). A 1:6 dilution with ultrapure water was performed for measurements.


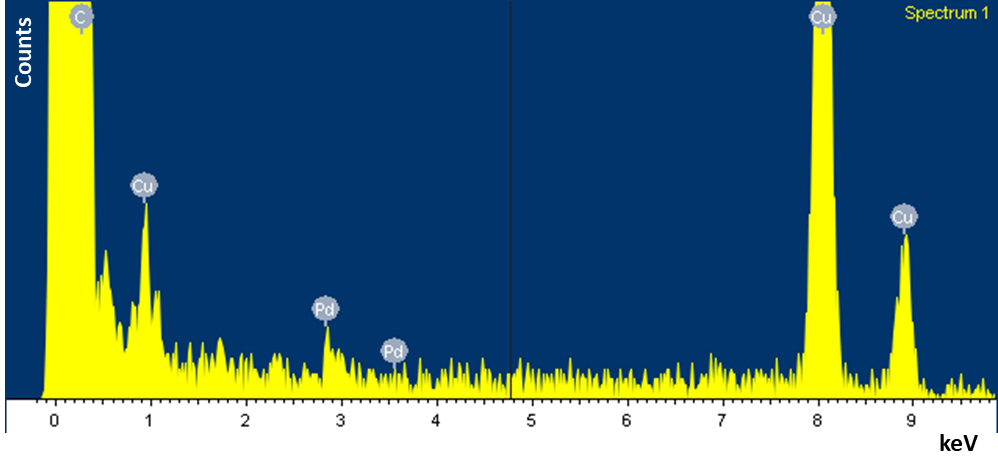


**Figure S2**. Elemental characterization of PdNCs by EDX. The spectrum collects the elemental composition at selected areas for the purified PdNCs (synthesized at 50ºC with 0.36 M NaBH_4_).

**A)**


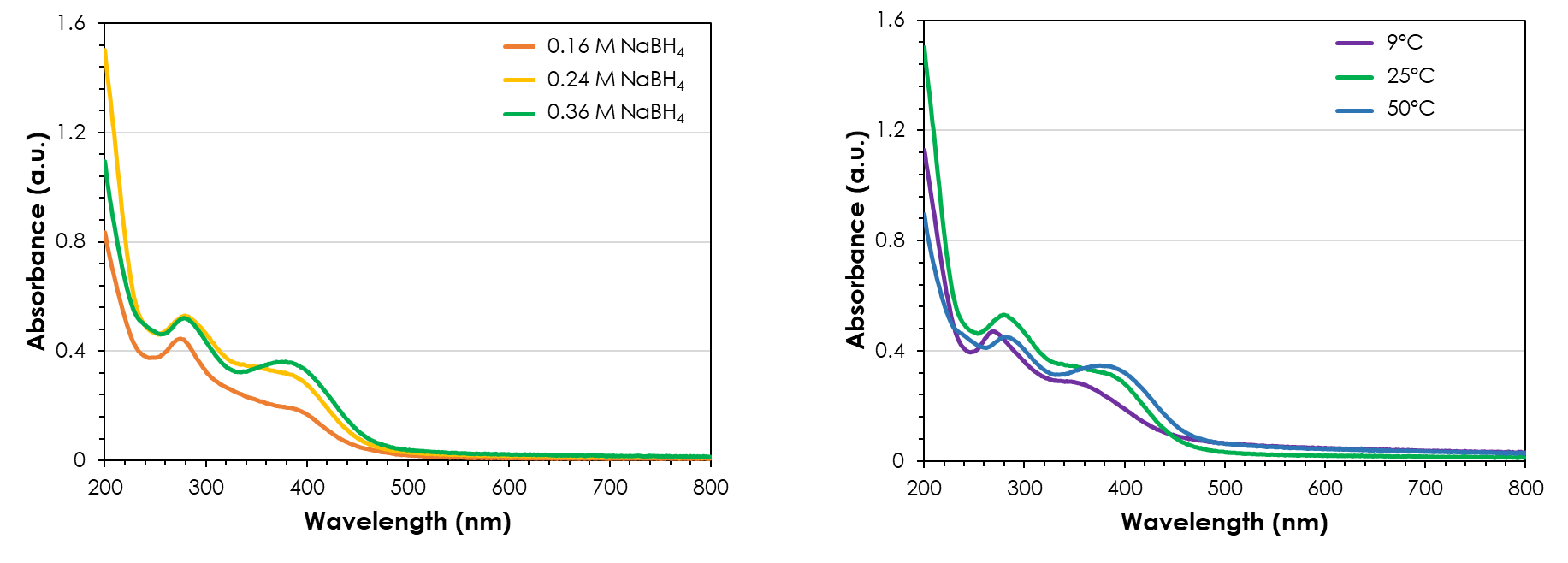


**B)**


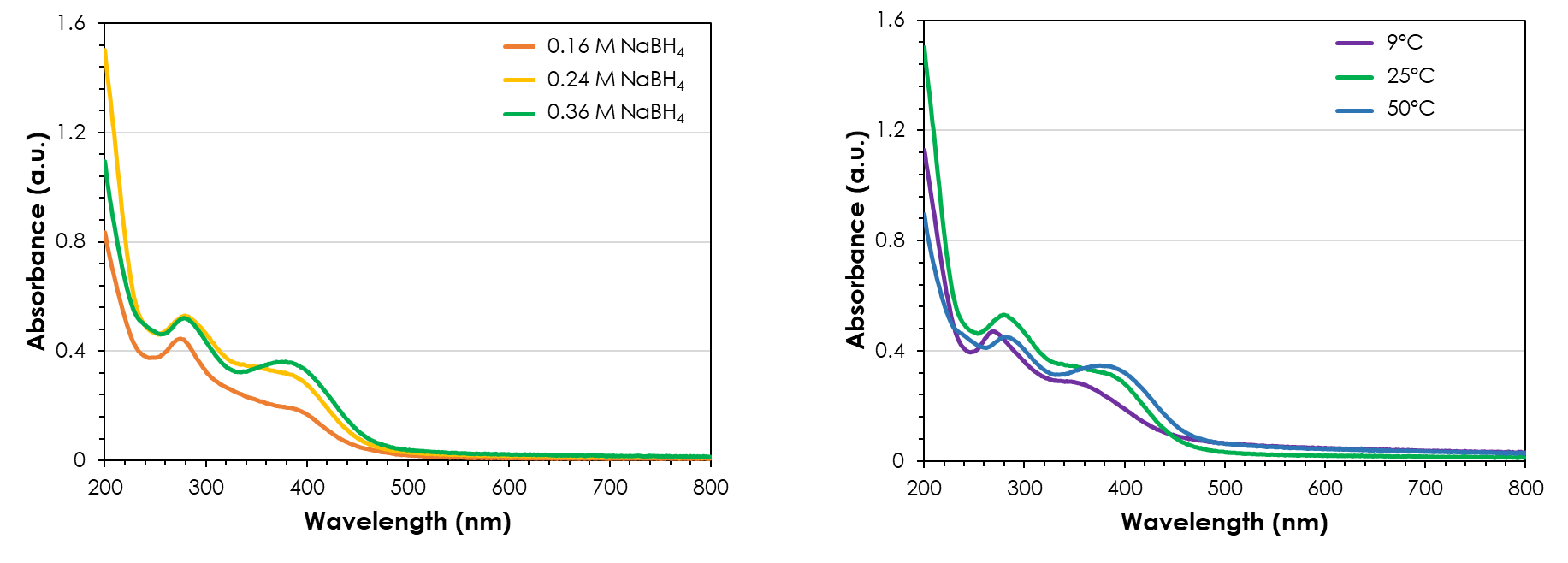


**Figure S3**. Absorbance spectra obtained for the purified PdNCs solutions synthesized at different concentrations of the reducing agent (0.16 M, 0.24 M and 0.36 M NaBH_4_) and temperatures (9ºC, 25ºC and 50ºC). A) Influence of NaBH_4_ concentration (at 25ºC), and B) Influence of the temperature (using 0.36 M NaBH_4_). A 1:10 dilution with ultrapure water was performed in all cases for measurements.

Concerning the characterization of the solution containing the synthesized PdNCs, it is necessary to calculate both the number of Pd atoms per NC and the Pd concentration in the suspension [1-4]. For the calculation of Pd atoms, it is first necessary to determine the crystal structure of PdNCs by SAED analysis. Moreover, the shape and size of PdNCs were obtained through HR-TEM measurements. Next, , considering that a face-centered cubic cell consists of 4 atoms, the mass of a unit cell can be calculated as 7.07·10^-22^ g·cell^-1^. Considering the Pd density (12.023 g·cm^-3^), each unit cell has a volume of 5.88·10^-23^ cm^3^·cell^-1^. Taking into account that PdNCs have a spherical shape (obtained by HR-TEM measurements), their volume can be then calculated from their experimental diameter (2.49 ± 0.02 nm). By dividing the volume of a PdNC by the volume of a unit cell, the number of cells per NC can be determined. Finally, multiplying the number of cells by the number of Pd atoms in a cell yields the number of Pd atoms per NC. On average, experimental results showed that the PdNCs contained 550 Pd atoms per NC.

In order to obtain the concentration of PdNCs, Pd was determined by ICP-MS after an acid digestion of the purified synthesized PdNCs suspensions. The concentration of Pd obtained was 0.405 ± 0.008 mM. Then, this Pd concentration was divided by the amount of Pd atoms that make up each NC, finding a value of 0.74 µM of PdNCs.

**Evaluation of PdNCs Catalytic Activity on HER**

Figure S4 shows the voltammograms obtained by CV (sweeping the potential between -1.5 V and +1.5 V) for the procedural blanks and the purified PdNCs solutions (with a 1:25 dilution in the different media).

1. **0.1 M NaOH**


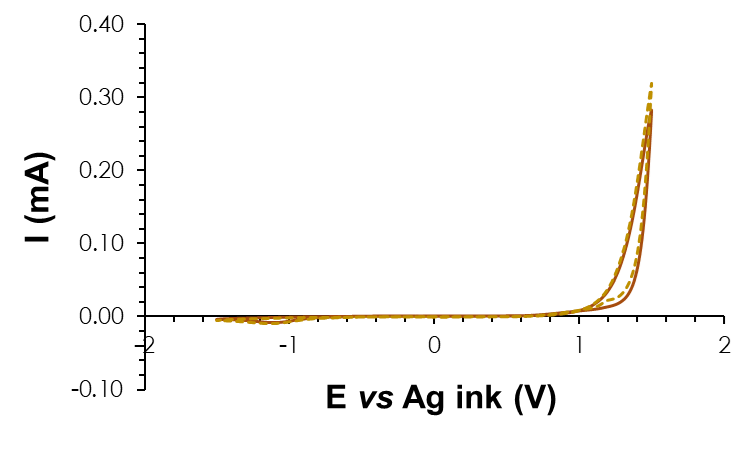


1. **0.1 M PB pH 7.6**


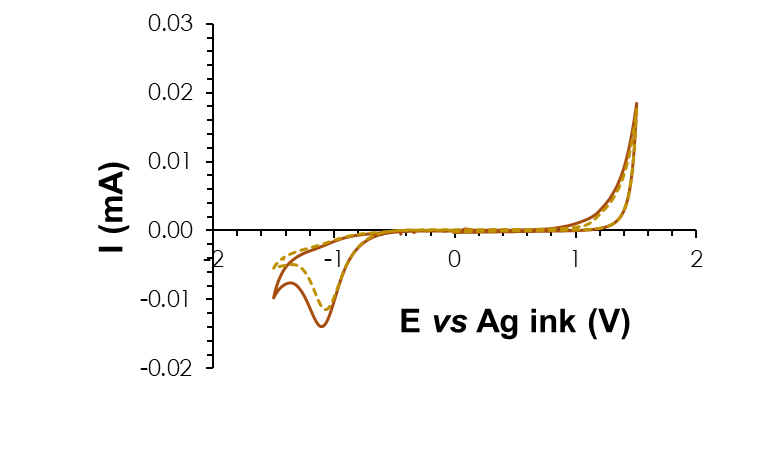


1. **0.1 M HNO_3_**

**
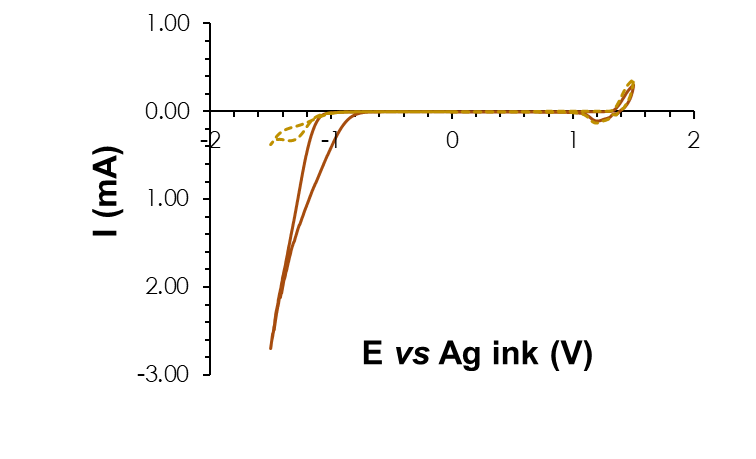
**

**Figure S4**. Voltammograms obtained by cyclic voltammetry (scanning the potential between -1.5 and +1.5 V) for the procedural blanks (NaOH, phosphate buffer or HNO_3_; dotted line) and the purified PdNCs solution (straight line) with a 1:25 dilution with the corresponding media to evaluate the effect of the pH. A) 0.1 M NaOH, B) 0.1 M PB pH 7.6, and C) 0.1 M HNO_3_.

**Selection of the Optimal Ab:NC Ratio in the PdNCs Immunoprobe and Optimization of the Competitive Immunoassay**

For the calculation of the real number of PdNCs per Ab (i.e., the immunoprobe stoichiometry), Figure S5 collects the relationship obtained between the concentration of Pd (expressed as pmoles) and the concentration of GFAP (in pmoles).


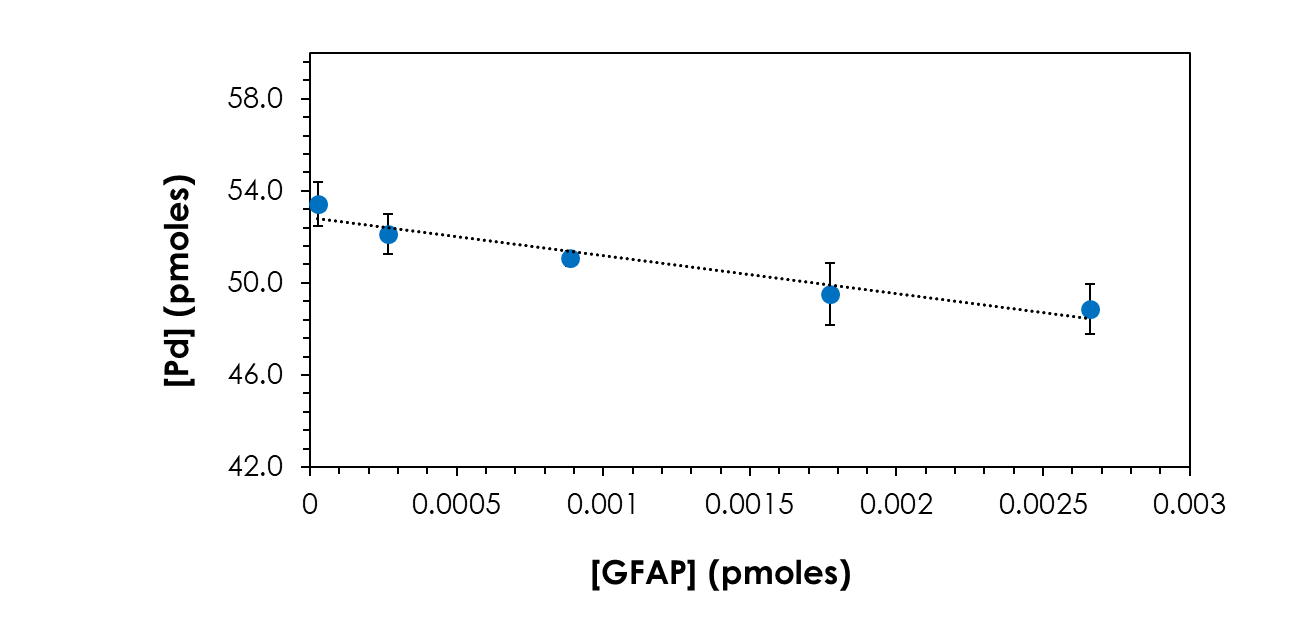


**Figure S5**. Determination of the Ab:PdNCs immunoprobe stoichiometry through a competitive immunoassay. Calibration graph obtained by ICP-MS representing the concentration of Pd vs. the concentration of GFAP. Uncertainties represent the standard deviations of the mean of three independent measurements.

**REFERENCES**

[1] Cruz-Alonso M, Trapiella-Alfonso L, Costa-Fernández JM, Pereiro R, Sanz-Medel A (2016) Functionalized gold nanoclusters as fluorescent labels for immunoassays: Application to human serum immunoglobulin E determination. Biosensors and Bioelectronics 77:1055–1061

[2] Valencia E, Cruz-Alonso M, González-Iglesias H, Fernandez B, Pereiro R (2019) Fluorescent silver nanoclusters as antibody labels for the determination of complement factor H in human serum. Microchimica Acta 186:429

[3] Lores-Padín A, Cruz-Alonso M, González-Iglesias H, Fernández B, Pereiro R (2019) Bimodal determination of immunoglobulin E by fluorometry and ICP-MS by using platinum nanoclusters as a label in an immunoassay. Microchimica Acta 186:1-10.

[4] Menero-Valdes P, Lores-Padín A, Fernández B, González-Iglesias H, Pereiro R (2022) Iridium nanoclusters as highly sensitive-tunable elemental labels for immunoassays: Determination of IgE and APOE in aqueous humor by inductively coupled plasma-mass spectrometry. Talanta 244:123424.
